# A closer look at the antibiotic‐resistant bacterial community found in urban wastewater treatment systems

**DOI:** 10.1002/mbo3.589

**Published:** 2018-02-27

**Authors:** Amir H. Tehrani, Kimberley A. Gilbride

**Affiliations:** ^1^ Department of Chemistry and Biology Ryerson University Toronto ON Canada; ^2^ Ryerson Urban Water Ryerson University Toronto ON Canada

**Keywords:** antibiotic resistance, microbial communities, tetracycline, wastewater

## Abstract

The conventional biological treatment process can provide a favorable environment for the maintenance and dissemination of antibiotic‐resistant bacteria and the antibiotic resistance genes (ARG) they carry. This study investigated the occurrence of antibiotic resistance in three wastewater treatment plants (WWTP) to determine the role they play in the dissemination of ARGs. Bacterial isolates resistant to tetracycline were collected, and tested against eight antibiotics to determine their resistance profiles and the prevalence of multiple antibiotic resistance. It was found that bacteria resistant to tetracycline were more likely to display resistance to multiple antibiotics compared to those isolates that were not tetracycline resistant. Polymerase chain reaction (PCR) was used to identify the tetracycline resistance determinants present within the bacterial communities of the WWTPs and receiving waters, and it was found that ARGs may not be released from the treatment process. Identification of isolates showed that there was a large diversity of species in both the tetracycline‐resistant and tetracycline‐sensitive populations and that the two groups were significantly different in composition. Antibiotic resistance profiles of each population showed that a large diversity of resistance patterns existed within genera suggesting that transmission of ARG may progress by both horizontal gene and vertical proliferation.

## 1 INTRODUCTION

The use of antibiotics in the treatment of infectious diseases is crucial for the protection of public health. However, the significant increase in the use and misuse of antimicrobials drugs, both in clinical and agricultural settings, has contributed to a corresponding increase in the concentration of compounds found in waste streams and the environment in general (World Health Organization and Who, 2001). In urban settings, humans contribute to most of the pharmaceutical waste that ends up in domestic sewers that eventually is transported to the wastewater treatment plant (WWTP). Wastewater treatment includes the removal of particulate matter and degradation of organic and inorganic contaminants; however, conventional wastewater treatment facilities have not been designed to remove emerging contaminants during treatment (Zhang & Li, 2011). Furthermore, removal rates depend heavily on the operating conditions of the plant (Guerra, Kim, Shah, Alaee, & Smyth, 2014; Lishman et al., 2006). Concurrently, daily use of pharmaceuticals for medical treatment and agricultural uses results in pseudopersistent concentrations of these drugs in the secondary process of the WWTPs. Surveys of Canadian WWTPs have shown that antibiotics can frequently be detected in effluents (Guerra et al., 2014; Miao, Bishay, Chen, & Metcalfe, 2004), and can enter the environment through discharges from the wastewater treatment process.

The increase in antibiotic waste released into municipal wastewater has coincided with an increase in the prevalence of resistance genes in wastewater treatment processes. Both antibiotic‐resistant bacteria (ARB) and antibiotic resistance genes (ARGs) have been found in wastewater samples from China, Japan, Germany, Portugal, and the US (Arkansas, Colorado, Louisiana, Michigan) among others (Bouki, Venieri, & Diamadopoulos, 2013; Everage, Boopathy, Nathaniel, LaFleur, & Doucet, 2014; Goñi‐Urriza, Capdepuy, Arpin, Raymond, & Pierre Caumette, 2000; Kümmerer, 2009; Munir, Wong, & Xagoraraki, 2011; Novo, André, Viana, Nunes, & Manaia, 2013; Pruden, Pei, Storteboom, & Carlson, 2006; Schwartz, Kohnen, Jansen, & Obst, 2003; Zhang et al., 2015). Limited data is available on the abundance and identification of ARB and ARGs in WWTPs in Canada (Rahube & Yost, 2010), and it is not clear what role the WWTPs play in discharging ARGs into the natural environment along with treated effluent (Bouki et al., 2013). Concerns about WWTPs acting as sites for the transfer and evolution of ARGs prompts the following questions: What is the prevalence of ARGs in WWTPs in a large Canadian urban metropolis? Are ARGs escaping from WWTPs and contaminating downstream water bodies? And what are the ARBs in the WWTP that carry the resistance genes? Are ARGs in wastewater carried as single entities or they are part of multiple gene mobility factors and how are they inherited?

Although antibiotic resistance and corresponding gene determinants are ubiquitous in the environment, WWTPs are considered “hotspots” for horizontal gene transfer between bacteria due to their high‐nutrient and high‐density load (Rizzo et al., 2013). Furthermore, the subinhibitory concentrations of antibiotics, like those observed in WWTPs, have been shown to increase the frequency of transfer of resistance genes (Finley et al., 2013). The ability of WWTPs to act as an ideal environment to promote gene transfer between bacteria endorses the potential for the increased occurrence of ARGs within the bacterial population and the potential for resistance genes to be transferred from indigenous populations to pathogens or from one pathogen to another (Ham et al., 2012). Overall, the WWTP may accelerate the evolutionary timeline of ARGs by enhancing the mobilization of environmental resistance genes into clinical isolates (Marti, Variatza, & Balcazar, 2014). Studies examining the relative abundance of ARGs in water and biofilm samples collected downstream of WWTP sites suggest that effluent discharges could be the source of ARGs in the environment (Marti et al., 2014; Storteboom, Arabi, Davis, Crimi, & Pruden, 2010).

Tetracycline is an antibiotic that has been used extensively in human and veterinary medicine for decades. Although its usage in human treatment has decreased in recent years, its consumption in agricultural and animal husbandry settings is still widespread. Resistance to tetracycline is due to numerous genes that code for one of three mechanisms: efflux pumps, ribosomal protection proteins, or enzyme degradation. Many of these genes are found on mobile genetic elements that carry resistance to other antibiotics and/or metals (Roberts, 2005; Zhang, Zhang, & Ye, 2011). With more than 40 determinants identified with the code for resistance to tetracycline (Van Hoek et al., 2011) and the genetic basis of the resistance being well established, molecular methods can be used to track the identification and location of the determinants in populations (Peak et al., 2007).

The aim of this study was to devise a characterization strategy to permit the investigation of the prevalence and fate of ARGs in WWTPs and demonstrate the interaction between antibiotic‐sensitive and ‐resistant populations within a community. We hypothesize that by using both molecular‐ and culture‐based techniques, we can identify suitable donors and recipients from each respective population that can serve as ideal candidates to understand the fate of ARGs in wastewater communities. Since culture‐dependent methods may isolate only a small percentage of the overall bacterial population and culture‐independent methods are limited in their ability to identify the antibiotic resistance isolates or determine multiple antibiotic resistance in the population, both culture‐dependent and ‐independent methods were employed in this study to minimize the limitations of either approach. The objectives were to isolate tetracycline‐sensitive and tetracycline‐resistant bacteria from multiple urban WWTPs to determine the frequency of resistance, and whether selection for a single resistance increased the likelihood that isolates carried multiple resistance; to identity isolates from the two populations to determine whether genus identity was correlated with antibiotic resistance phenotypes profiles; to track tetracycline resistance genes to determine if WWTP are seeding natural environments; and to perform hierarchical cluster analysis on the resistance profiles as a way to detect gene dissemination. Although culture‐independent techniques such as next‐generation sequencing can provide more information with regard to the entire population, they lack the ability to identify which of the members carry individual genes. Our combined methods of characterization enable downstream population investigations that monitor the proliferation and transfer of mobile genetic elements among native members in a given environment, and the fate of those elements after transfer.

## 2 EXPERIMENTAL PROCEDURE

### 2.1 Sample sites and collection

Wastewater grab samples were collected from the secondary treatment process (approximately one third through the process) of three wastewater treatment plants in the Toronto area: North Toronto WWTP, Ashbridges Bay WWTP, and the Humber WWTP. The North Toronto plant treats approximately 34,000 cubic meters per day with excess wastewater being diverted to Ashbridges Bay via gravity. Ashbridges Bay WWTP is the largest wastewater treatment plant in Toronto and has a capacity to treat 818,000 cubic meters of wastewater per day (Wastewater, 2016). Its final effluent is discharged approximately 1 km out into Lake Ontario on the east side of the city. Humber WWTP currently processes 473,000 cubic meters of wastewater per day and discharges its final effluent into Lake Ontario on the west side of the city.

All WWTPs were sampled at least on three separate occasions (0.5 L each time) and at multiple times through the year. All samples were transported to the laboratory and processed on the day they were collected. In total, nine samples were processed (as outlined below) and 168 isolates were selected to represent the diversity and abundance of the isolates for further analysis. The results from each location were combined to minimize differences due to weather, incoming sewage consistency, etc., and reflect the situation at that location.

Environmental samples (1 L) were collected from the shoreline of Lake Ontario in two locations—each one approximately 1 km downstream of the WWTP discharge site. In this case, the samples were filtered and only processed for DNA extraction.

### 2.2 Isolation of tetracycline (*Tet*)‐resistant and sensitive bacteria

Aliquots from each sample were plated in triplicates on Reasoner's 2A agar (R2A) with and without tetracycline (16 μg/ml). Plates were incubated at room temperature for up to 3 days and CFUs were counted. Culturable aerobic plate counts were used to determine the frequency of tetracycline‐resistant bacteria at each site. From the plates, a total of 168 bacterial isolates were randomly selected to maximize diversity of the samples and regrown for further analysis.

### 2.3 Antibiotic testing of isolates

The isolates were divided into those that were sensitive to tetracycline (59) and those that were resistant (109) and tested for antibiotic susceptibility to eight antibiotics using the standard Kirby–Bauer disk diffusion method and the protocol provided by Becton Dickinson BBI sensi‐disk antimicrobial susceptibility text manual, except that isolates were tested on R2A agar instead of Mueller–Hinton since environmental isolates prefer a less rich media. Laboratory strains of *Escherichia coli* (DH5α) and *Pseudomonas putida* (BBC 443 and ATCC 12633) were used as controls to test whether changing the media to R2A agar affected the inhibition zone diameters set out in the manual. The isolate's antibiotic profile was classified as sensitive, intermediate, or resistant using the diameters set out for heterotrophic bacteria by the manufacturer (BD‐Canada). The antibiotics tested were ampicillin (10 μg), chloramphenicol (30 μg), ciprofloxacin (5 μg), gentamicin (10 μg), kanamycin (30 μg), streptomycin (10 μg), sulfamethoxazole/trimethoprim (23.75/1.25 μg), and tetracycline (30 μg). The following antibiotics were selected to represent some of the major antibiotic groups and based on the availability: penicillin (ampicillin), aminoglycosides (gentamicin, kanamycin, streptomycin), chloramphenicol (chloramphenicol), tetracyclines (tetracycline), fluoroquinolone (ciprofloxacin), and co‐trimoxazole (sulfamethoxazole/trimethoprim).

The percentage of multiple ARB at each location was determined. An isolate was considered to be multiple antibiotic resistant (MAR), if it was found to be resistant to three or more antibiotics (Krumperman, 1983).

### 2.4 Antibacterial resistance index (ARI)

The antibacterial resistance index is used for analyzing the prevalence of bacterial‐resistant determinants in a population at a specific location. The following formula was used to calculate the ARI:

ARI=A/NY,

where *A* is the total number of resistant determinates recorded in the population, *N* is the number of isolates in the population, and *Y* is the total number of antibiotics tested (Mohanta & Goel, 2014).

### 2.5 DNA extraction

DNA was extracted from both pure isolates and activated sludge samples using the MoBio UltraClean Soil DNA Extraction Kit (MoBio Laboratories Inc., Carlsbad, CA), following the manufacturer's protocol. Certain steps in the protocol were modified to obtain better DNA yield which included increasing the bead‐lysis step for certain cultures from 10 min to 20 min. Furthermore, the elution step incubation period was increased by an additional 5 min. The concentration and purity of DNA was determined via a nanophotometer and gel electrophoresis. DNA was stored at −20°C until needed for polymerase chain reaction (PCR) amplification and sequencing.

### 2.6 Determination of tetracycline resistance determinant

A qualitative PCR assay was performed to determine which of the seven tetracycline resistance determinants (Tet B Tet C, Tet G, Tet M, Tet Q Tet W, and/or Tet X) were present in the WWTPs community extracts. The genes and the primers are shown in Table 1. Each reaction was tested alongside an appropriate positive and negative control to ensure the validity of the PCR protocol. Positive controls were plasmids obtained from M.C. Roberts (University of Washington) containing the appropriate gene to Tet B, Tet C, Tet G, Tet M, Tet Q, and Tet W, and DNA from strains containing Tet X provided by G. Vora (Naval Research Base, Washington) as shown in Table 1. Genomic DNA extract of an *E. coli* DH5α laboratory strain with no tetracycline resistance was used as the negative control for this assay.

**Table 1:** A list of PCR primers used for tetracycline resistance determinants and the 16S rRNA gene

Gene	Primer	Sequence (5’–3’)	Amplicon size (bp)	Annealing temperature (^o^C)	Resistance mechanism	Positive control	Ref
Tet B	Tet B f Tet B r	TTGGTTAGGGGCAAGTTTTG GTAATGGGCCAATAACACCG	659	TD (65–55)	Efflux pump	*Escherichia coli* HB101 (pRT11)	Ng, Martin, Alfa, and Mulvey (2001)
Tet C	Tet C f Tet C r	CTTGAGAGCCTTCAACCCAG ATGGTCGTCATCTACCTGCC	418	TD (65–55)	Efflux pump	*E*. *coli* DO‐7 (pBR322)	Ng et al. (2001)
Tet G	Tet G f Tet G r	GCTCGGTGGTATCTCTGCTC AGCAACAGAATCGGGAACAC	468	TD (65–55)	Efflux pump	TOPO10	Ng et al. (2001)
Tet M	Tet M f Tet M r	GTGGACAAAGGTACAACGAG CGGTAAAGTTCGTCACACAC	406	TD (65–55)	Ribosomal protection protein	*E*. *coli* DH1 (pACYC177)	Ng et al. (2001)
Tet Q	Tet Q f Tet Q r	ATCGGTATCAATGAGTTGTT GACTGATTCTGGAGGAAGTA	40	50	Ribosomal protection protein	pNFD 13.2	(Bartha, Sóki, Urbán, & Nagy, 2011)
Tet W	Tet W f Tet W r	GAGAGCCTGCTATATGCCAGC GGGCGTATCCACAATGTTAAC	168	TD (65–55)	Ribosomal protection protein	pIE1120 (pGEM‐TW)	Aminov, Garrigues‐Jeanjean, and Mackie (2001)
Tet X	Tet X f Tet X r	TTAGCCTTACCAATGGGTGT CAAATCTGCTGTTTCACTCG	223	55	Degradation enzyme	DNA	Bartha et al. (2011)
16S rRNA	U341 f U758 r	CCTACGGGAGGCAGCAG CTACCAGGGTATCTAATCC	500	55	NA	Any bacteria	

TD, touchdown PCR (initial temperature = 65°C and final temperature = 55°C); NA, not applicable.

Each PCR reaction was performed in 25 μl reactions containing 50 ng of template DNA, 0.5 μmol/L of forward and reverse primers, 3.44 μg BSA, 200 μmol/L dNTPs, Taq buffer (10 mmol/L Tris‐HCl pH 9.0, 50 mmol/L KCl, 1.5 mmol/L MgCl_2_) with 1.25 units Taq (New England BioLabs, Pickering, ON, Canada). Touchdown‐PCR (TD‐PCR) was performed on Tet B, Tet C, Tet G, Tet M, and Tet W due to high efficacy. The first step involved sample denaturation at 96°C for a duration of 5 min followed by thermocycling at 94°C for 1 min. An initial annealing temperature of 65°C was decreased by 1°C for every cycle for a total of 10 cycles with an elongation time of 3 min at 72°C. Annealing temperature of 55°C was used for an additional 20 cycles. The reaction composition of Tet Q, and Tet X were similar to the previously listed reaction, except that 0.7 μmol/L forward and reverse primers were used. The following thermocycling settings was used for Tet Q: initial denaturation at 94°C for 5 min, 94°C denaturation for 30 s, annealing temperature at 50°C for 30 s, and elongation temperature at 72°C for 1.5 min for a total of 30 cycles. Lastly, the following thermocycling settings was used for Tet X: initial denaturation at 94°C for 2.5 min, 94°C denaturation for 15 s, annealing temperature at 55°C for 30 s, and elongation temperature at 72°C for 30 s for a total of 35 cycles. Four microliters of the PCR products was run on a 1% agarose gel (stained with SYBR Safe) at 100 V for 25–30 min via gel electrophoresis (Invitrogen, Burlington, ON, Canada).

### 2.7 DNA sequencing and phylogenetic analysis

Isolates for sequencing were selected from each WWTP in order to provide the same representation in the isolate pool as seen in the community pool as determined by morphology and antibiotic profiles. Of the 168 original isolates, DNA was successfully extracted from 90 isolates (45 tet‐sens and 45 tet‐res). The 16S rRNA gene was then amplified by PCR using the forward primer U341 F and reverse primer U758 R (Table 1). The reaction composition and thermocycler setting that was used to carry out the 16S rRNA gene amplification was identical to that of the TD‐PCR mentioned above. DNA sequencing of the 16S rDNA PCR products were performed at the ACGT (Toronto, ON, Canada) with a Sanger sequencing system. A single consensus sequence was generated and edited from the forward and the reverse nucleotide sequences using Sequence Scanner v1.0 (Applied Biosystems, 2005).

The sequences obtained were imported into NCBI Nucleotide‐BLAST database to determine the identity of each isolate. Once the species were identified, appropriate type strains were selected from NCBI database and both were imported into Molecular Evolutionary Genetic Analysis (MEGA 7.0) software. By using the Clustal W alignment tool, the sequences were aligned among each other and the fragment lengths were accommodated to the shortest sequence (>400 bp). Both dendrograms were constructed separately to address the tetracycline‐resistant and tetracycline‐sensitive isolates. Dendrograms were created using the neighbor‐joining statistical method, and bootstrap values were generated from 500 replications.

The Shannon–Weaver Diversity Index (Fedor & Spellerberg, 2013) was used to test the evenness and diversity in each of the populations, and the Dice coefficient (Dice, 1945) was used to determine the similarity of the two populations. To identify distinct patterns of resistance among the isolates, hierarchical cluster analysis was performed. The inhibition zones obtained from the antimicrobial disk susceptibility test were categorized into nominal values based on their phenotypes (resistant, susceptible, and intermediate). The nominal values were then imported into IBM SPSS statistics program version 23.0 to generate the clusters using Square Euclidean distance and the Ward method. The patterns of resistance observed in each cluster were organized based on the number of isolates demonstrating the same type of resistance for a given antibiotic. If 75% or more of the isolates had an identical phenotype, they were categorized accordingly as S (susceptible) or R (resistant). If less than 75% of the isolates in a given cluster demonstrated a particular phenotype, they were categorized as Variable Resistance (V^x^‐%, where x is the phenotype demonstrated by the majority of the cultures, followed by the percentage).

## 3 RESULTS AND DISCUSSION

Both tetracycline‐resistant and ‐sensitive cultures were isolated from wastewater samples from three urban WWTPs in Toronto and were characterized using culture‐dependent, culture‐independent, and statistical techniques. The advantage of combining these methods of characterization allows the collection of information about the community as a whole with respect to the array of resistance determinants that are contained within that community.

### 3.1 Antibiotic resistance

Tetracycline resistance genes have been shown to be widespread in the microbial community in hospital and urban WWTPs. Furthermore, the percent of bacteria isolates exhibiting antibiotic resistance within WWTPs was found to be greater than that found in the natural environment (Iwane, Urase, & Yamamoto, 2001; Rizzo et al., 2013). Auerbach, Seyfried, and McMahon (2007) used culture‐independent methods to show that tetracycline resistance genes were more abundant in WWTPs than in natural lake samples and that Tet Q was found to be highest in influent and Tet G to be highest in activated sludge. Moreover, ARBs and ARGS have been found to be released from WWTP in the effluent and biosolids generated during the treatment process (Munir et al., 2011).

**Figure 1:**
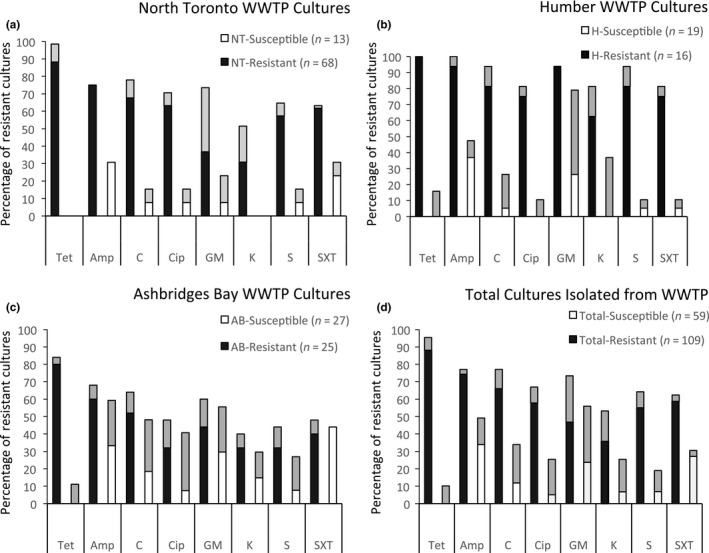
Antibiotic resistance profiles of tetracycline‐sensitive and tetracycline‐resistant isolates from three urban wastewater treatment plants; (a) Isolate profiles from North Toronto WWTP (b) Isolate profiles from Humber WWTP, (c) Isolate profiles from Ashbridges Bay WWTP, and (d) cumulative total from all three locations. The black bars show the percentage of resistant isolates and the white bars represent the percentage of sensitive isolates. The gray bars present the percentage of isolates exhibiting intermediate resistance. Tet, tetracycline; Amp, ampicillin; C, chloramphenicol; Cip, ciprofloxacin; GM, gentamicin; K, kanamycin; S, streptomycin; SxT, sulfomethoxozle/trimethylprim

In this study, bacterial isolates were collected from three WWTPs in a large urban area by spread plating activated sludge samples on R2A plates with and without selective antibiotic. Fifty‐nine tetracycline‐sensitive isolates representing different morphotypes were selected for further analysis, 13 were from the North York plant, 22 were from the Humber plant, and 29 were from the Ashbridges Bay plant. It was found that when these isolates were tested for their resistance to eight antibiotics, many were found to have resistance to one or more of the antibiotics (Figure 1). Antibiotic resistance to each of the eight antibiotics was found in all the plants albeit at varying levels of resistance. Overall, it was found that 33%–37% of the isolates were resistant to ampicillin, 5%–18.5% were resistant to chloramphenicol, 0%–7.6% resistant to ciprofloxacin, 26%–29 % were resistant to gentamicin, 0%–14.8% resistant to kanamycin, 5.2%–7.7 % resistant to streptomycin, and 5%–44% were resistant to sulfamethoxazole /trimethoprim. Although 30% of the isolates were not resistant to any of the antibiotics tested, 13.6% were found to be resistant to three or more antibiotics and therefore considered to have MAR.

Regardless of the WWTP sampled, the resistance to ampicillin appeared to be quite consistent with approximately one‐third of the bacteria carrying resistance to the β‐lactam antibiotics. Other studies have found that ampicillin resistance varies from 3.3% to 42% (Rizzo et al., 2013) and is among the most common resistance found. Ampicillin resistance is mediated by the *bla* (TEM‐1) gene that can be carried on a transposon or plasmid, and contributes substantially to the spread of the antibiotic resistance determinant among bacterial populations (Uyaguari, Fichot, Scott, & Norman, 2011).

Resistance to sulfamethoxazole/trimethoprim was also found to be high and bacteria have been found to remain resistant to this drug even in the absence of selective pressure (Gao et al., 2012). Resistance to kanamycin was only observed in the Ashbridges Bay WWTP isolates and resistance to ciprofloxacin was only observed in the Humber and Ashbridges Bay plant isolates, although several additional isolates displayed intermediate resistance to these antibiotics. Overall, the isolates that were collected without antibiotic selection carried a wide diversity of resistance (Figure 1).

The percent of tetracycline‐resistant culturable bacteria in the WWTPs was determined by plating samples on R2A plates supplemented with tetracycline (16 μg/ml) and expressing the number of isolates that grew on tetracycline plates as a percentage of the total number of bacteria that grew on plates with no antibiotic. Each WWTP was sampled three times and the percentage tetracycline‐resistant isolates varied from as little as 0.13 % to as high as 7.18 % of the total culturable population. Several of the isolates that grew on the tetracycline selective plates later showed only intermediate resistance when tested in the antibiotic disk test, probably due to the fact that the disks contained 30 μg, while the selective plates had only contained 16 μg/ml tetracycline. Overall, it was found that 0.94% of the culturable bacterial population from Ashbridges Bay, 1.84% from Humber, and 3.66 % from North Toronto were resistant to tetracycline (Figure 1).

One hundred and nine of these isolates were then tested for their resistance to the additional seven antibiotics, 68 from the North Toronto, 25 from Ashbridges Bay, and 16 from the Humber WWTP. Overall, it was found that 75%–94% of the tetracycline‐resistant isolates were also resistant to ampicillin, 52%–81% were resistant to chloramphenicol, 32%–75% resistant to ciprofloxacin, 37%–94% were resistant to gentamicin, 31%–63% resistant to kanamycin, 32%–81% resistant to streptomycin, and 40%–75 % were resistant to sulfamethoxazole/trimethoprim (Figure 1). In total, 78% were considered to be MAR (Table 2).

**Table 2:** The MAR and ARI scores for the tetracycline‐sensitive and tetracycline‐resistant isolates from each of the urban WWTPs

WWTP	Tet profile (*n*)	MAR	ARI
North Toronto	Tet‐sensitive (13)	7.7	0.11
	Tet‐resistant (68)	80.9	0.60
Ashbridges Bay	Tet‐sensitive (29)	22.2	0.21
	Tet‐resistant (25)	56.0	0.47
Humber	Tet‐sensitive (22)	5.3	0.10
	Tet‐resistant (16)	100	0.83
Total	Tet‐sensitive (64)	13.6	0.14
	Tet‐resistant (109)	78.0	0.60

Comparison of the levels of resistance between isolates selected as tetracycline‐sensitive and tetracycline‐resistant suggests that selection for a single‐resistant determinant makes it more likely for the isolates to have additional resistances, probably because resistance genes are often found clustered on mobile genetic elements that can be transferred to other bacteria (Wellington et al., 2013).

The ARI scores (Table 2) of the tetracycline‐sensitive and tetracycline‐resistant isolates were calculated. An ARI value above 0.2 indicates that isolates are exposed to selectivity due to the presence of contaminants such as antibiotics (Mohanta & Goel, 2014). Since selective pressure can promote dissemination of the resistance determinants, a population with a high ARI score would have more members carrying resistance genes that were likely to proliferate or transfer resistance genes to other organisms. In our case, the ARI scores for the tetracycline resistance population in all three WWTP (0.60, 0.47, and 0.83) were up to eight times higher than those in the tetracycline‐sensitive population (0.11, 0.21, and 0.10), suggesting that dissemination of ARGs had occurred and that mobile genetic elements carrying multiple gene resistances were likely present (corresponds with high MAR).

Both the Humber and North Toronto plants had higher ARI values than Ashbridges Bay possibly because Ashbridges Bay collects a much greater volume of water including storm water that may dilute the antibiotic concentrations from municipal sources thereby lowering the selection pressure.

### 3.2 Diversity and abundance of species

Forty‐five tetracycline‐sensitive and 45 tetracycline‐resistant isolates were successfully sequenced and identified (Table 3). All the isolates presented 16S rRNA gene sequence had similarity values higher than 95 % with the type strain of a validly named species and were therefore considered members of that genus. Most (67%) of the tetracycline‐sensitive isolates were identified as *Acidovorax, Acinetobacter, Aeromonas, Flavobacterium*, and *Pseudomonas*, while most (56%) of the tetracycline‐resistant isolates were found to be *Chryseobacterium, Microbacterium*,* Stenotrophomonas*, and *Variovorax*, showing that the composition of the populations were dominated by different genera. The Shannon–Weaver Index calculation confirmed that both the tetracycline‐sensitive population (0.64) and the tetracycline‐resistant population (0.66) contained a large amount of diversity, and a Dice coefficient calculation (0.20) indicated that the populations did not have a significant overlap in composition, suggesting that they were distinctly different from one another.

**Table 3:** Identities of the tetracycline‐resistant and tetracycline‐sensitive WWTP isolates

Genus	Tet‐resistant – *n* (%)	Tet‐sensitive – *n* (%)
*Achromobacter*	2 (4.4)	0
*Acidovorax*	1 (2.2)	6 (13.3)
*Acinetobacter*	3 (6.7)	10 (22.2)
*Aeromonas*	1 (2.2)	5 (11.1)
*Chryseobacterium*	6 (13.3)	0
*Enterobacter*	0	2 (4.4)
*Escherichia*	2 (4.4)	0
*Exiguobacterium*	0	3 (6.7)
*Flavobacterium*	0	4 (8.9)
*Herminiimonas*	0	1 (2.2)
*Klebsiella*	1 (2.2)	0
*Microbacterium*	6 (13.3)	1 (2.2)
*Morganella*	1 (2.2)	0
*Providencia*	1 (2.2)	0
*Pseudomonas*	2 (4.4)	5 (11.1)
*Riemerella*	0	2 (4.4)
*Serratia*	3 (6.7)	0
*Sinorhizobium*	0	1 (2.2)
*Staphylococcus*	1 (2.2)	1 (2.2)
*Stenotrophomonas*	9 (20.0)	1 (2.2)
*Variovorax*	4 (8.9)	1 (2.2)
*Xanthomonas*	2 (4.4)	1 (2.2)
*Yersinia*	0	1 (2.2)
Total (*n*)	45	45

In terms of dissemination of tetracycline resistance determinants, we were able to find both sensitive and resistant variants of some genera; however, seven genera (*Enterobacter*,* Exiguobacterium*,* Flavobacterium*,* Herminiimonas*,* Riemerella*,* Sinorhizobium*,* and Yersinia*), representing 14 isolates (31%), were only found among the tetracycline‐sensitive strains (Table 3). Interestingly, many of the possible pathogenic genera (*Escherichia* and *Serratia*) were only found in the tetracycline‐resistant population, confirming the potential concern of antibiotic resistance dissemination among pathogens in wastewater treatment communities. Although some pathogens were found in the tetracycline‐sensitive population, none was represented in that population. This observation confirms that antibiotic resistance in pathogens is quite widespread in WWTPs.

The uniqueness in composition of the two populations possibly reflects the limitation of antibiotic gene dissemination among some bacterial genera. Although the culturable population represents only a fraction of the total community, it may indicate that not all bacteria are capable of carrying or expressing every antibiotic resistance determinant. Furthermore, it appears that horizontal gene transfer may be restricted to certain members of the overall bacterial community. Further characterization of these isolates will determine if they share common genetic elements that can be used to carry antibiotic genes.

### 3.3 Dissemination of antibiotic genes into the environment

**Figure 2:**
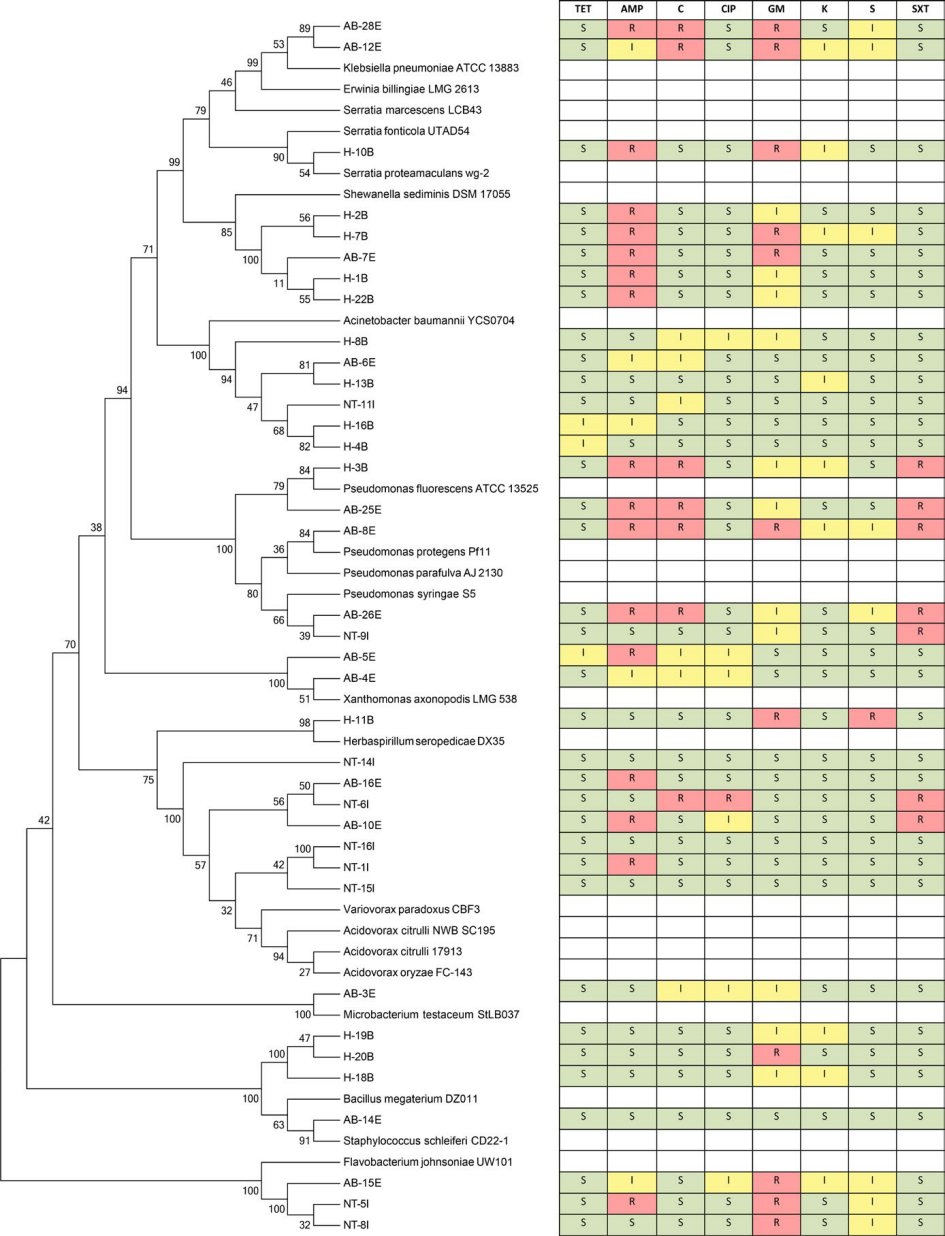
Dendrogram of the alignment of the 16S rRNA partial sequences of various tetracycline susceptible isolates to known type strains with their corresponding antibiotic resistance profiles. Antibiotics used: TET, tetracycline (30 μg); AMP., ampicillin (10 μg); C, chloramphenicol (30 μg); CIP, ciprofloxacin (5 μg); GM, gentamicin (10 μg); K, kanamycin (30 μg); S, streptomycin (10 μg); and SXT, sulfamethoxazole/trimethoprim (23.75/1.25 μg); R, resistance; I, intermediate; S, sensitive

**Figure 3:**
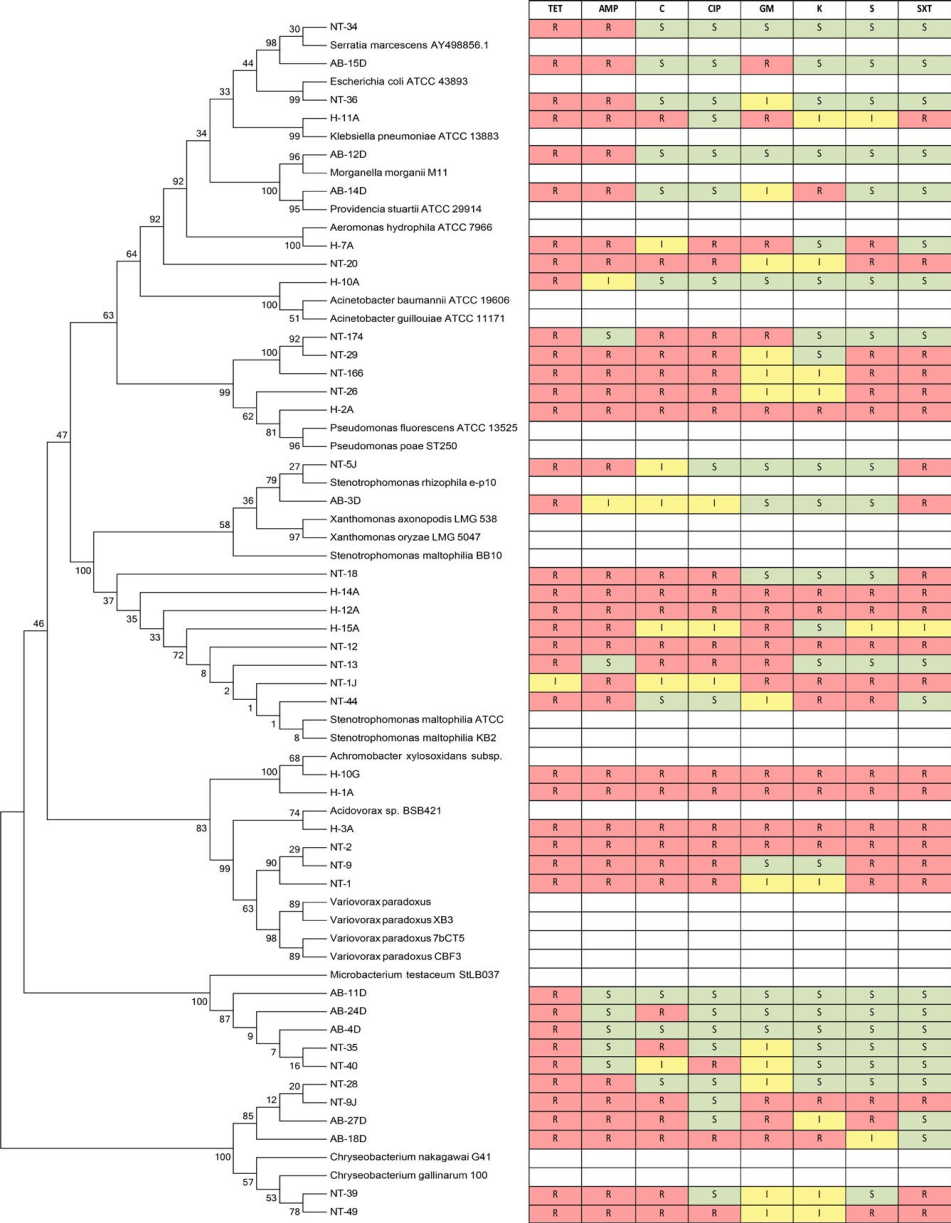
Dendrogram of the alignment of the 16S rRNA partial sequences of various tetracycline‐resistant isolates to known type strains with their corresponding antibiotic resistance profiles. Antibiotics used: TET, tetracycline (30 μg); AMP, ampicillin (10 μg); C, chloramphenicol (30 μg); CIP, ciprofloxacin (5 μg); GM, gentamicin (10 μg); K, kanamycin (30 μg); S, streptomycin (10 μg); and SXT, sulfamethoxazole/trimethoprim (23.75/1.25 μg); R, resistance; I, intermediate; S, sensitive

Antibiotic resistance was analyzed in conjunction with the phylogenetic data using a cluster analysis to compare the antibiotic resistance determinant patterns within each genera cluster and throughout each population (Figures 2 and 3). Differences in antibiotic resistance patterns can result from the ecology and physiology of the bacteria and may suggest distinct modes and mechanisms of resistance acquisition. In one case, there were no distinct resistance patterns associated with any of the genera clusters. For example, within the tetracycline‐sensitive population, the *Acinetobacter* cluster contained seven different antibiotic profiles, none of which were more dominant than the other or more prevalent in any one of the WWTPs. This possibly suggests that individual isolates had acquired their resistance genes independently of others. Since our identification did not identify specific species within each cluster, it is possible that antibiotic resistance patterns may emerge at the species level (Vaz‐Moreira, Nunes, & Manaia, 2011).

In the tetracycline resistance population, the *Stenotrophomonas* cluster (Figure 3) not only contained isolates from both the North Toronto and Humber plant that presented the same pattern (resistant to all eight antibiotics), but also contained isolates with different patterns within and between the different WWTPs. Again this suggests that individual isolates may have acquired resistance genes independently of each other or such that these determinants are not actively expressed despite their presence. Nevertheless, on some occasions, different isolates of the same genera, isolated from either the same or different WWTPs, yielded the same antibiotic resistance pattern. In general, it was observed that members of the same genera did not necessarily share common antibiotic resistance profiles. Moreover, it was not possible to establish a relationship between the resistance phenotype and the site of isolation. However, the absence of any patterns across any of the parameters—genera, location, or antibiotic resistance—suggests the relevance of population dynamics for the hypothetical dissemination of resistance. To evaluate whether vertical and horizontal gene transfer is the major process for dissemination of antibiotic resistance within WWTPs, a deeper analysis to include multiple isolations of the same species from different time points is required.

### 3.4 Dissemination of antibiotic genes into the environment

Antibiotic resistance bacteria and genes not removed during WWT process could potentially be disseminated into the environment downstream of the discharge pipe (Rahube & Yost, 2010). Although ARB are seldom released from the WWT process, ARGs can escape removal (Pruden et al., 2006). If ARGs are present on small genetic elements, they may be able to pass through the discharge process and be available for uptake (via transformation) by bacteria present downstream of the plants. ARGs themselves have been recognized as emerging contaminants, independent of their bacterial carriers (Storteboom et al., 2010). Therefore, identifying the contribution source of the ARGs in the downstream water sources can help to determine how much the WWTPs contribute to urban water ARG contamination.

Two wastewater flow pathways were investigated. The North Toronto Plant discharges its effluent by gravity to the Ashbridges Bay plant, which after treatment releases the effluent into Lake Ontario on the east side of the city (LOS1). The Humber plant discharges its final effluent directly into Lake Ontario on the west side of the city (LOS2). Identification of the tetracycline determinants may assist in monitoring the dissemination of tetracycline resistance and the evolution of gene exchange. Previous sampling showed that tetracycline‐resistant bacteria could be isolated from all five locations (data not shown); however, whether the source of the tetracycline‐resistant bacteria in the lake were due to inherent resistance levels or due to acquisition of ARGs from the WWTP discharge was unknown.

Overall, the percentage of tetracycline‐resistant bacteria in the lake was lower than in the WWTP (data not shown) which could be expected since a large dilution effect must be taken into account when the discharge is released into the lake body. In order to determine if the antibiotic genes in the WWTP are indeed escaping to the lake, seven of the tetracycline resistance determinants were monitored using PCR primers to the seven genes to create ARG gene profiles of the bacterial populations within the three urban WWTPs and the receiving waters. The seven genes used were Tet B, Tet C, Tet G, Tet M, Tet Q, Tet W, and Tet X.

Table 4 shows the detection of each of the genes in the five locations. Tet C, Tet Q and Tet X were found in all locations suggesting that these gene determinants are ubiquitous in Toronto water, whereas Tet B was not detected in any of the locations. Tet G was found in the North Toronto plant and Ashbridges Bay plant but not in the Humber plant; however, this determinant was absent from all lake samples at both locations. Likewise, Tet M was found in all three WWTPs although not in all samples from the Ashbridges Bay plant and was not in the downstream water body. This evidence suggests that the WWTP process may effectively remove some ARGs from the effluent before their release. Interestingly, however, Tet W was found in all three WWTPs and in the downstream lake water but only in the water column and not in the sediment. The detection of Tet W in the water column and not in the sediment perhaps represents a transient location for the ARG where the determinant has not been deposited into the lake in high enough concentration or over enough time to allow the determinant to be deposited into the sediment or be picked up by a bacterium that eventually settles into the sediment. Moreover, it could not be determined whether the determinant was indigenous to the lake water or had come from being released from the WWTP although the same result was observed in both wastewater pathways. Further investigation of the presence of this determinant in water sources upstream of the plants could help to determine if the WWTP contributed to the presence of Tet W in the lake water or whether this determinant is indigenous to the lake.

**Table 4:** The detection of tetracycline gene determinants in metagenomics DNA in the two wastewater flow pathways

	Gene determinant						
	Tet B	Tet C	Tet G	Tet M	Tet Q	Tet W	Tet X
Pathway 1							
North Toronto	−	+	+	+	+	+	+
↓							
Ashbridges Bay	−	+	+	+/−	+	+	+
↓							
Lake Ontario (east) water column or	−	+	−	−	+	+	+
Lake Ontario (east) sediment	−	+	−	−	+	−	+
Pathway 2							
Humber	−	+	−	+	+	+	+
↓							
Lake Ontario (west) water column or	−	+	−	−	+	+	+
Lake Ontario (west) sediment	−	+	−	−	+	−	+

### 3.5 Hierarchical cluster analysis of antibiotic profiles

The antibiotic profile patterns of 160 isolates were compared using hierarchical cluster analysis to determine if common patterns could be distinguished. After analysis, the 160 profiles were clustered into 10 major patterns (Table 5). There are two ways of interpreting the data obtained through the cluster analysis. The first is to recognize R (resistance) and S (susceptibility) patterns in each cluster that are over the 75% cut‐off. Possible genetic elements could be present within the cluster populations that confer resistance to certain groups of antibiotics within the populations. In other words, one or more mobile elements may be responsible for conferring similar patterns of resistance in each cluster. Because of this phenomenon, it is possible that the proliferation of multiple antibiotic resistance carrying elements have spread throughout various members of the population, thus giving them a similar resistance pattern. For example, by observing clusters 9 and 10, there are 25 isolates in cluster 10 with various morphologies but an identical resistance phenotype. Similarly, cluster 9 also has a similar pattern of resistance with the exception of a high number of intermediate levels of resistance to gentamicin and kanamycin. It is possible that the resistance patterns observed for both clusters 9 and 10 may be due to a similar mobile element that lacks the gentamicin and kanamycin resistance genes in cluster 9; or alternatively, isolates in cluster 10 have an additional element with resistance genes to these two antibiotics.

**Table 5:** Distinct patterns of resistance of the isolates obtained through hierarchical cluster analysis

Cluster	Tet	Amp	C	Cip	GM	K	S	SXT	Number of isolates in each cluster
1	S	S	V^S^‐68%	S	V^S^‐64%	S	S	S	28
2	V^S/R^‐46%	R	S	S	V^I/R^‐46%	S	S	S	13
3	S	V^S/R^‐42%	S	S	R	V^S^‐58%	V^I^‐50%	S	12
4	R	S	V^S^‐50%	V^S^‐71%	V^S^‐64%	S	S	S	14
5	S	V^S^‐58%	V^S^‐58%	V^I^‐58%	S	S	S	R	12
6	R	R	V^R^‐54%	V^R^‐45%	S	S	S	V^R^‐63%	11
7	R	R	V^S/R^ ‐36%	V^S^‐54%	R	V^R^‐73%	V^R^‐73%	S	11
8	V^R^‐58%	R	R	S	V^R^‐58%	V^R^‐42%	V^I^‐42%	R	12
9	R	R	R	R	V^I^‐50%	V^I^‐50%	R	R	22
10	R	R	R	R	R	R	R	R	25

Tet, tetracycline; Amp, ampicillin; C, chloramphenicol; Cip, ciprofloxacin; GM, gentamicin; K, kanamycin; S, streptomycin; SXT, sulfamethoxazole/trimethoprim, S‐(≥75% Susceptible), R‐(≥75% Resistant), V^S^‐% (<75% susceptible as majority), V^R^‐% (<75% resistant as majority), V^I^‐% (<75% intermediate as majority), V^S/R^‐% (<75% susceptible and resistant in equal distribution), V^I/R^‐% (<75% intermediate and resistant in equal distribution).

The second way of interpreting the data is to pay attention to the variable resistance (V^R^) phenotypes across the clusters. Resistances to certain antibiotics across most clusters show various degrees of susceptibility (resistant, susceptible, and intermediate). As a result, only the prominent phenotype is indicated in Table 5 (e.g., V^S^‐58% indicates that 58% of the isolates in this cluster was susceptible to the antibiotic). The variable phenotypes in a given cluster introduces discrepancies across isolates , making it difficult to categorize the susceptibility of the cultures. Meanwhile, these variable phenotypes could indicate the possibility of mobile‐mediated‐resistance genes being present in some cases and absent in others. For example, cluster 8 contains a population where the majority are resistant to ampicillin, chloramphenicol, and sulfamethoxazole/trimethoprim, yet remain variable for tetracycline, gentamicin, kanamycin, and streptomycin. This pattern of resistance could be the result of a mobile genetic element carrying resistance to aminoglycosides and/or tetracycline in some of the isolates within this cluster, while absent in others. Cluster 7 also shares a similar concept for resistance. The majority of the isolates in cluster 7are resistant to tetracycline, ampicillin, and gentamicin, yet remain variable for chloramphenicol, ciprofloxacin, kanamycin, and streptomycin. It is possible for a mobile genetic element carrying resistance for the natural aminoglycosides to be present among some of these isolates with a possibility of also carrying resistance to chloramphenicol and ciprofloxacin.

After examining the collected data, it is difficult to differentiate the root cause of these patterns, and whether they are caused by a single mobile genetic (with insertions or deletions) or by numerous/combinations of genetic elements (plasmid, transposons, or chromosomal). However, these patterns do provide the incentive for investigating wastewater cultures to determine which genetic elements are responsible for resistance to the targeted antibiotics and to determine whether they can horizontally be transferred, particularly to pathogenic microbes.

Overall, bacterial isolates were collected from the three urban WWTPs and found to have multiple resistances to eight antibiotics. Bacteria that carried a single resistance to tetracycline were found to be more likely to have resistance to three or more antibiotics than those isolates that were not tetracycline‐resistant. This suggests that resistance could be acquired as a cassette containing several determinants or that a single determinant could code for a mechanism that can offer resistance to several different antibiotics simultaneously. A more diverse tetracycline determinant library was seen in the WWTP than in the receiving waters, indicating that ARGs may be removed during the treatment process (Table 4). However, sampling of receiving waters at a later date will determine if determinants only seen in the WWTP eventually appear in the receiving waters. Identification of isolates showed that there was a large diversity of species in both the tetracycline‐resistant and tetracycline‐sensitive populations and that the two groups had unique compositions suggesting that antibiotic resistance determinants may be more likely to be present in some strains than in others (Table 3). Furthermore, a large diversity of antibiotic resistance patterns existed within the genera of each population, suggesting that transmission of ARG within the WWTP process may happen by several different mechanisms. Last, in future studies, it would be valuable to identify which mobile genetic elements are carried by these bacterial cultures. Not only would it provide an insight on how mobile genetic elements may proliferate in a population, but also will identify the members involved in their transfer. By characterizing the population using our combination of methods, we were able to link genotypes to specific communities and phenotypes to specific community members. It allowed us to gain a deeper understanding on how gene transfer may or may not occur in highly dense populations and who maybe the possible donors and recipients.

## CONFLICT OF INTEREST

None declared.
